# Odontogenic myxoma with rare sunburst periosteal reaction: report of two cases and review of the literature

**DOI:** 10.1007/s11282-026-00925-7

**Published:** 2026-04-22

**Authors:** Anne Evelyn Oliveira Moura, José Erivaldo da Silva Mendes, Elaine Judite de Amorim Carvalho, Fábio Abreu Alves, André Caroli Rocha, Danyel Elias da Cruz Perez

**Affiliations:** 1 Oral Diagnosis Department, Semiology and Oral Pathology Areas, Piracicaba Dental School, University of Campinas (UNICAMP), Piracicaba, São Paulo, Brazil; 2 Oral Pathology Unit, Department of Clinical and Preventive Dentistry, Universidade Federal de Pernambuco (UFPE), Recife, Pernambuco Brazil; 3 Stomatology Department, A. C. Camargo Cancer Center, São Paulo, Brazil

**Keywords:** Qdontogenic myxoma, Sunburst periosteal reaction, Differential diagnosis, Diagnostic imaging

## Abstract

Odontogenic myxoma (OM) is a benign tumor originating from odontogenic ectomesenchyme. While this tumor displays imaging variability, the sunburst appearance is uncommon and can resemble malignant lesions. In this report, we present two cases of OM exhibiting an imaging sunburst appearance, besides a literature review. A 35-year-old woman and a 21-year-old woman presented with mandibular swelling. Imaging exams revealed extensive radiolucent/hypodense images, with the presence of a sunburst periosteal reaction. The main diagnostic hypothesis for both cases was osteosarcoma. Incisional biopsies were performed, with definitive diagnosis of OM. The first patient underwent simple surgical excision, while the second underwent en bloc resection. Neither patient experienced recurrence during the follow-up period of three and one year after treatment. Odontogenic myxoma should be considered in the differential diagnosis of jaw lesions presenting a sunburst periosteal reaction, as this atypical imaging pattern may mimic aggressive or malignant conditions.

## Introduction

 Odontogenic myxoma (OM) is a benign odontogenic tumor characterized by sparse spindle- or stellate-shaped cells within a myxoid stroma [1]. The tumor is more common in the second and third decades of life, but it can occur at any age. A female predilection is observed and the mandible is the most common site, especially the posterior region [1, 2]. OM is the third most common odontogenic tumor [1]. Clinically, OM manifests as an infiltrative and locally invasive neoplasm [2].

OM presents different radiographic features [3]. Most lesions are multilocular with internal septa [2], producing characteristic radiographic patterns, such as “soap bubbles” (rounded compartments of varying sizes), “honeycomb” (numerous small, relatively uniform compartments resembling a honeycomb), or “tennis racket” (thin, straight or angular septa creating sharp, triangular or square compartments, resembling a tennis racket) [3, 4]. Rarely, a “sunray” or “sunburst” appearance may be observed in OM [4–8]. This pattern results from perpendicular stretching and subsequent ossification of Sharpey’s fibers connecting the periosteum to the bone, generating a periosteal reaction. This radiographic finding is most commonly observed in malignant tumors, particularly osteosarcoma [3]. Thus, this report describes two cases of OM presenting a sunburst pattern, and review of the literature.

## Case reports

### Case 1

A 35-year-old woman presented with a chief complaint of mobility of the left mandibular second molar, which had been present for three months. This tooth was submitted to endodontic treatment three years ago. No extraoral alterations were observed. Intraoral examination revealed a mandibular lingual cortical swelling and the presence of a gingival nodular lesion in the region of the left mandibular second molar.

**Fig. 1:**
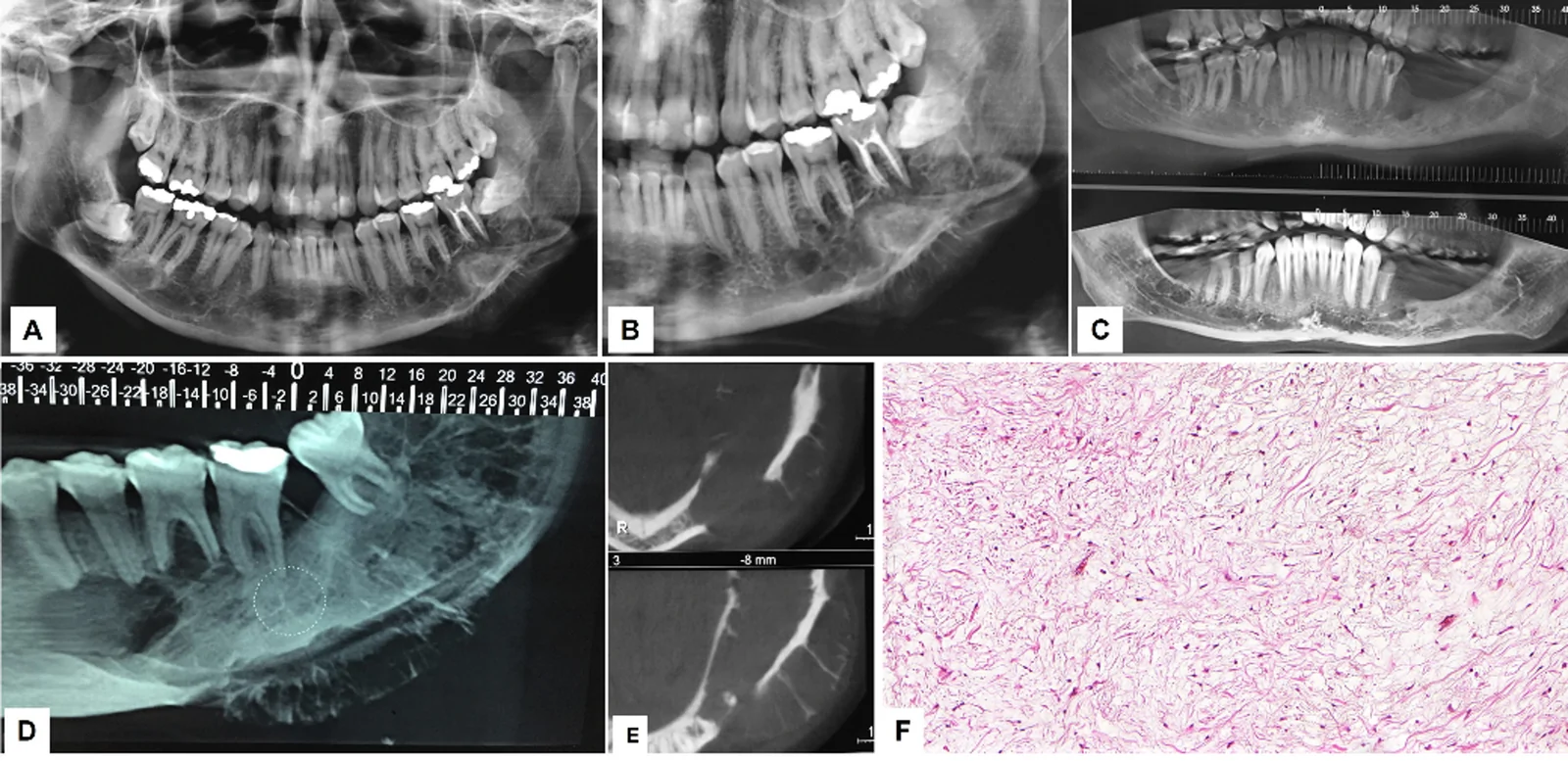
**a** Case 1 - Panoramic radiography exhibiting radiolucent, multilocular and ill-defined image located in the left mandibular body and ramus. **b** Case 1 – Linear septa originating from the mandible’s inferior border arranged in a radiating pattern, resembling a sunburst appearance **c** Case 1 – no signs of recurrence were observed after three years of treatment **d** Case 2 - A destructive, mixed, multilocular image involving the left mandibular body, angle and ramus, a sunburst periosteal reaction. Root resorption was also evident **e** Case 2 – Cone beam computed tomography, axial view, displaying expansion, thinning and ruptures of the vestibular and lingual cortices, with periosteal reaction resembling sunburst **f** Histopathological analysis demonstrated fusiform and stellate cells within a loosely arranged myxoid tissue. No cellular atypia or mitoses were found (original magnification, hematoxylin-eosin, x100)

Panoramic radiography revealed a radiolucent, multilocular and ill-defined image located in the left mandibular body and ramus. Linear septa originating from the inferior border of the mandible were observed, arranged in a radiating pattern, resembling a sunburst appearance (Fig. 1a, b). The diagnostic hypothesis was a malignant neoplasm, mainly osteosarcoma. The panoramic radiograph was obtained at an external diagnostic center; therefore, detailed acquisition parameters were unavailable in the patient records and could not be retrieved.

Under local anesthesia, an incisional biopsy was made. The histopathological analysis revealed the presence of fusiform and stellate cells within a loose myxoid tissue, alongside a slightly fibrous stroma. No cellular atypia or pleomorphism was noted and odontogenic epithelial islands were not identified. No immunohistochemical analysis was performed. The definitive diagnosis was OM. Surgical excision of the lesion followed by curettage was performed. After three years of follow-up, no signs of recurrence were observed (Fig. 1c).

### Case 2

A 21-year-old woman was referred for diagnosis of a painful facial asymmetry, with five months of duration. Intraoral examination revealed a left mandibular swelling with a hardened consistency. The overlying mucosa appeared normal.

On panoramic radiography, an ill-defined, multilocular, and mixed image was observed, located in the left mandibular body and ramus. Cone beam computed tomography (CBCT), on panoramic reconstruction, showed a destructive, mixed, multilocular image involving the first left mandibular premolar region. The image extended to the angle and the entire left mandibular ramus, resulting in root resorption and perforation of the base of the mandible. Additionally, a sunburst periosteal reaction was evident (Fig. 1d). Both coronal and axial views revealed expansion, thinning and ruptures of the buccal and lingual cortices, accompanied by a periosteal reaction resembling sunburst (Fig. 1e). The main diagnostic hypothesis was a malignant neoplasm, especially osteosarcoma. The panoramic radiograph and CBCT examinations were obtained at external diagnostic centers; therefore, detailed acquisition parameters were unavailable in the patient records and could not be retrieved.

An incisional biopsy was performed under local anesthesia. Microscopic findings were similar to case 1 (Fig. 1f). A block surgical resection was performed. After one year of follow-up, no signs of recurrence were observed.

### Differential diagnosis

The most critical differential diagnosis in this context is osteosarcoma, a primary malignant bone neoplasm that commonly presents with an aggressive periosteal reaction, ill-defined margins, and rapid clinical progression. Gnathic osteosarcoma typically shows a combination of osteolytic and osteoblastic changes resulting in a mixed imaging appearance, besides irregular bone formation, periodontal ligament space widening, and a destructive growth pattern that progresses over a short period [9]. In contrast, OM, despite its locally infiltrative behavior, usually demonstrates a slow-growing course, and absence of malignant osteoid formation, with radiographic findings that may include multilocular radiolucency and residual trabeculae rather than true tumor bone production [1]. Other entities that may show a sunburst or spiculated periosteal reaction include Ewing sarcoma, chondrosarcoma, metastatic disease, and aggressive osteomyelitis. These conditions are often associated with systemic symptoms, soft-tissue mass formation, or clinical signs of infection, which are missing in OM [10].

Although a sunburst periosteal reaction is classically associated with malignant bone tumors, its presence may be present in benign lesions. Bone benign lesions, such as odontogenic myxoma, may mimic aggressive malignancies on imaging [11, 12].

Regarding the histopathological aspects, according to the WHO Classification of Head and Neck Tumours, the most significant differential diagnosis for odontogenic myxoma is the dental follicle or developing dental papilla, which are histologically identical but clinically and radiographically distinct. In lesions with a more collagenized stroma, additional differential diagnoses include chondromyxoid fibroma, odontogenic fibroma, and low-grade myxofibrosarcoma [1]. However, in the two reported cases, the stroma was predominantly myxoid, without a collagenized stroma. Careful assessment of growth pattern, clinical behavior, and correlation with histopathological findings is essential to avoid misdiagnosis and inappropriate management.

## Discussion

The radiographic characteristics of OM exhibit considerable variation [3, 5–7]. Nevertheless, the sunburst periosteal reaction is rare in OM [13]. It is characterized by a rapid expansion of the lesion, in which the periosteum does not have enough time for the formation of an adequate new layer, resulting in the development of perpendicular fibers [14]. To the best of our knowledge, only 21 cases of OM with this imaging appearance have been reported, based on a literature search performed in PubMed/MEDLINE using the following search strategy, with no language or date restrictions: *(myxoma OR myxomas OR “odontogenic myxoma” OR “odontogenic myxomas”) AND (“sun rays” OR “sun ray” OR sunbeams OR sunburst OR sunrays OR sun-rays OR sun-ray OR sunray OR “cone beam computed tomography” OR “computed tomography” OR radiographic OR radiological OR radiography OR panoramic radiography OR sun) AND (jaw OR jaws OR mandible OR maxilla OR odontogenic)* [3–8, 13, 15–26] (Table 1).

The sunburst imaging appearance is commonly associated with aggressive lesions, including metastases [27]. A study assessing periosteal reactions and intraosseous lesions, 25% of cases exhibiting a spiculated-type reaction—of which the sunburst pattern is a subtype—were associated with malignant lesions [28]. Although the sunburst pattern was observed in Case 1 on panoramic radiography, this imaging modality has been shown to have low sensitivity for detecting periosteal reactions [29]. Therefore, when this radiographic feature is identified, the differential diagnosis should include more aggressive lesions, particularly primary or metastatic malignant neoplasms.

Odontogenic myxoma (OM) occurs more commonly in females, with a mean age of 28.6 years, similar to present cases, including those with a sunburst radiographic appearance (Table 1). In addition, the mandible is more frequently affected than the maxilla [2]. In the present cases, patients were treated with surgical excision. However, in previously reported cases, wide surgical resection was the most common treatment modality (38.1%). Furthermore, no recurrence was observed in 61.8% of patients, as observed in the current cases.

The diagnosis of OM must be confirmed by histopathological analysis. The two reported cases and those described in the literature showed fusiform and stellate cells, immersed in a myxoid tissue [3–8, 13, 15–26], essential characteristics described for OM [1]. Hence, there are no microscopic differences between OM with and without a sunburst appearance.

The treatment of OM may vary from enucleation or curettage to wide surgical resection [2]. Most cases with a radiographic appearance of sunburst, for which treatment information was available, were underwent to an en bloc resection/wide surgical resection [6–8, 15–19, 22, 26], similar to the case 2. In case 1, a surgical excision followed by curettage was performed. No recurrences were noted, as observed in the previously described cases exhibiting a similar imaging appearance [6–8, 13, 15–20, 24, 26]. Therefore, the sunburst imaging appearance apparently is not associated with increased potential for tumor recurrence.

In summary, OM exhibits varied imaging characteristics. Despite the infrequent sunburst appearance, it should be considered in the differential diagnosis of bone lesions showing this pattern. This reinforces the importance of clinicopathological and imaging correlation to guide biopsy planning and avoid overly aggressive management.

**Table 1:** Clinical and pathological findings of odontogenic myxomas published in the literature that exhibited a radiographic appearance resembling sunburst

Authors	Year	Age	Sex	Site	Treatment	Outcome (months)
Large; Niebel; Fredricks	1960	35	M	Mandible	Resection en bloc	No recurrence (48)
Schmidseder; Groddeck; Scheunemann	1978	34	NA	Mandible	Wide surgical resection	No recurrence (72)
Chuchurru et al.	1985	20	F	Mandible	Wide surgical resection	No recurrence (18)
Peltola et al.	1994	31	F	Maxilla	NA	NA
MacDonald Jankowski et al.	2004	34	F	Maxilla	NA	NA
Li et al.	2006	22	F	Mandible	Resection en bloc	No recurrence (84)
Thomas et al.	2011	27	F	Mandible	Wide surgical resection	No recurrence (24)
Zhang et al.	2014	28	M	Mandible	NA	NA
		27	M	Mandible	NA	NA
Guo et al.	2014	14	M	Mandible	Resection en bloc	No recurrence (30)
Dabbaghi et al.	2016	27	F	Mandible	Wide surgical resection	No recurrence (24)
Goel; Goel; Dinkar	2016	18	F	Mandible	Hemimandibulectomy	No recurrence (12)
Kidwai et al.	2016	28	M	Mandible	Surgical excision	No recurrence (NA)
Varun et al.	2016	35	F	Mandible	NA	NA
Wang et al.	2017	NA	NA	Maxilla	Wide surgical resection	No recurrence (NA)
		NA	NA	Mandible	Wide surgical resection	No recurrence (NA)
Garg et al.	2018	22	M	Mandible	No treatment	NA
Das et al.	2019	17	F	Mandible	Surgical excision	No recurrence (03)
Kaur et al.	2020	72	F	Mandible	No treatment	NA
White et al.	2020	34	M	Mandible	Wide surgical resection	NA
Abdi et al.	2023	17	F	Mandible	Wide surgical resection	No recurrence (12)

*M* male, *F* female, *NA* not avaliable

## Data Availability

No datasets were generated or analysed during the current study.
